# The must-have and nice-to-have experimental and computational requirements for functional frequency doubling deep-UV crystals

**DOI:** 10.1038/s41467-018-05411-1

**Published:** 2018-07-30

**Authors:** P. Shiv Halasyamani, James M. Rondinelli

**Affiliations:** 1 Department of Chemistry, University of Houston, 112 Fleming Building, Houston, TX 77204-5003 USA; 2 Department of Materials Science and Engineering, Northwestern University, Evanston, IL 60208-3108 USA

**Keywords:** Nonlinear optics

## Abstract

Inorganic materials exhibiting second-harmonic generation (SHG) are used to generate coherent radiation at wavelengths where solid-state laser sources are not available; that is, the deep UV (DUV) below 200 nm. Here, we describe the structure and optical property requirements that should be assessed to conclusively demonstrate the discovery of a functional DUV material for nonlinear optical (NLO) applications.

## Introduction

**Fig. 1:**
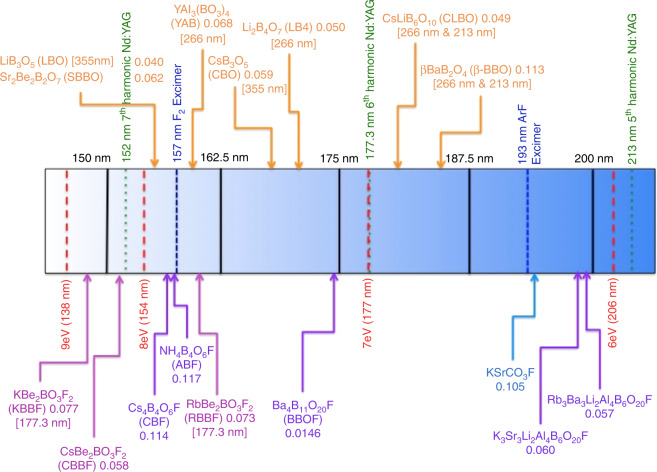
Deep-UV nonlinear optical materials. Deep-UV spectrum from ∼225 nm to 125 nm depicting the absorption edges of some NLO materials, along with excimer wavelengths F_2_ and ArF (blue dashed lines), 5th, 6th, and 7th harmonics of Nd:YAG: 1064 nm (green dotted line), and energies at 6, 7, 8, and 9 eV (red dashed lines). The arrows denote the absorption edge for each material, where the numbers in square brackets are the wavelengths where coherent radiation from a fundamental 1064 nm source is reported. The birefringence is given after the compound. See ref. ^10^ for the birefringence phase-matching condition expressions for uniaxial and biaxial crystals. Beryllium-containing borates exhibit absorption edges below 175 nm (7.08 eV), 147 nm (8.43 eV), and 160 nm (7.75 eV) for KBBF and RBBF respectively, as well as appropriate birefringence values, i.e., 0.080 (at 1064 nm for KBBF) and 0.073 (at 649.3 nm for RBBF) and nonlinear optical *d*_*ij*_ constants, 0.47 pm/V (KBBF) and 0.45 pm/V (RBBF). Adapted from ref. ^15^

Solid-state lasers capable of generating coherent radiation below 200 nm have uses in attosecond pulse generation, photoemission spectroscopy and photolithography. Currently, wavelengths below 193 nm (ArF excimer) (Fig. 1) can be reached by higher harmonic generation at the cost of efficiency. A 177.3 nm solid-state laser—the 6th harmonic of Nd:YAG (1064 nm)—could possibly replace excimer lasers if comparable efficiencies and performances could be obtained using a nonlinear optical (NLO) crystal through second-harmonic generation (SHG)^1^ through second-harmonic generation (SHG). Only two materials—KBe_2_BO_3_F_2_ (KBBF) and RbBe_2_BO_3_F_2_ (RBBF)—generate coherent radiation at 177.3 nm (Fig. 1)^2^ through cascaded frequency conversion. There are, however, two major issues with both materials: firstly, toxic BeO must be used in the synthesis, which is problematic as many nations limit exposure to beryllium. Secondly—and more importantly from a technological perspective—the materials exhibit a layered crystal structure, limiting single-crystal growth to at most 4 mm.

**Fig. 2:**
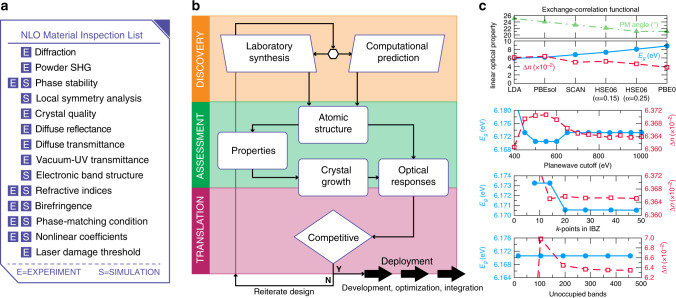
NLO Materials inspection. **a** The experiment (e) and simulation (s) must-have and like-to-have checklist itemizes important measurements and analyses to perform on a candidate compound; wherever feasible, both types of assessments should be performed. **b** Discovery workflow highlighting the main property assessments required to determine functionality—commercial viability—of new laboratory discovered or computationally predicted DUV NLO materials. Computational researchers can then facilitate the experimental laboratory discovery (hexagonal link) by providing confidence estimates to the prediction. **c** For materials predictions without experimental data, the quality of the results should be addressed by considering how different levels of theory, e.g., exchange-correlation functionals to DFT (upper panel), impact the predicted optical properties and phase stabilities (formation energies) of the crystal. Effect of the numerical accuracies of the plane-wave expansion, *k*-point sampling of the irreducible Brillouin zone (IBZ) and number of unoccupied bands, on the linear optical properties from DFT-PBEsol calculations for the experimental KBe_2_BO_3_F_2_ (KBBF) structure

The demonstration of a viable deep-UV (DUV) NLO material that generates coherent radiation at 177.3 nm requires multiple criteria to be satisfied simultaneously on either a newly synthesized material or on a chemically reasonable computationally predicted phase^3^. For the latter, careful attention is required as inaccurate property values can be obtained, as we show for KBBF (Fig. 2).

## Inversion symmetry

Second-order NLO phenomena may occur in noncentrosymmetric (NCS) materials^4^. The material must also allow phase-matching and should not crystallize in a cubic crystal class. Structural assessment can be done using diffraction-based methods and the stability and local symmetries of the NCS structure relative to a closely related centrosymmetric variant should be assessed using an electronic structure method, e.g., density functional theory (DFT), which would support the stability of the second-harmonic generation (SHG)-active NCS polymorph^5^. As a first step, powder SHG measurements (PSHG) can be used to corroborate the NCS crystal structure as $\chi _{ijk}^{\left( 2 \right)} \ne 0$ in NCS materials^6,7^. PSHG measurements as a function of particle size should be performed at 1064 nm and 532 nm and the PSHG intensities should be greater than KDP (at 1064 nm) and at least 0.25 × β-BBO (at 532 nm). If the intensities are substantially smaller than these, then efforts to grow large single crystals are unnecessary, as the material will have negligible DUV NLO properties.

## Crystal growth and quality

Large, high-quality single crystals of the material in question must be grown. For functional applications, crystals at least 5 mm along two dimensions should be reported. In addition, the crystals need to be processable such that useful crystallographic facets can be obtained, polished and indexed. With respect to quality, a rocking-curve measurement about a Bragg reflection with a full-width half-maximum of less than 100 arcsec (0.0278°)—ideally less than 50 arcsec (0.0139°)—must be shown. At the atomic scale, first-principles calculations can be used to assess surface energies of different crystal faces to understand factors affecting growth and processing.

## Optical responses

### Transparency

The optical responses are important properties to assess DUV NLO functionality. First, linear absorption should be measured; the material must be sufficiently transparent at both the fundamental and the doubled frequency. Diffuse reflectance and transmission measurements should be performed to assess whether the material is transparent down to at least 177.3 nm. Ideally, an electronic band gap of *E*_g_ > 7.08 eV (175 nm) is sought; however, as the gap increases typically the NLO coefficients decrease, a trade-off that needs to be circumvented. Diffuse reflectance measurements can determine the absorption edge above 200 nm, but vacuum-UV (VUV) transmission measurements on polished single crystals are required below.

Owing to advances in electronic structure methods, various levels of materials theory can predict optical gaps of materials, with DFT the most popular^8^. Many local and semi-local exchange-correlation functionals for DFT will, however, underestimate the experimental DUV band gaps (Fig. 2)—by up to 40%! For this reason, DFT calculations that include non-local exchange, incorporated in hybrid-functionals^9^ or more advanced methods, must be performed on any predicted compound, as no reference experimental gap is available to assess the DUV viability. When the experimental gap is known, it can be enough to correct the underestimated gap, but this can impact other linear responses (e.g., the birefringence).

### Phase-matching

SHG is most efficiently generated when the phase-matching conditions (PMC)^10^, *n*(2*ω*) = *n*(*ω*) are satisfied, where *n* is the refractive index. Therefore, at minimum, PMC requires that *n*_max_*(ω)*−*n*_min_*(*2ω) > 0. For crystals with normal dispersion, an indexed PMC can be achieved using the phase-matching (PM) angles over a specific range of wavelengths, i.e., a minimum PM wavelength < 177.3 nm is required.^11^ The wavelength range for the PMC is critically dependent on the birefringence: the larger the birefringence, the larger the phase-matching wavelength range. In most functional DUV NLO crystals, 0.07 ≲ Δ*n* ≲ 0.10 at 1064 nm. For small Δ*n*, the PMC is difficult to achieve in the DUV, whereas Δ*n* > 0.10 results in undesirable walk-off effects.

Experimental determination of the refractive indices, and birefringence, relies on high-quality single crystals. For an accurate measurement, either the minimum deviation technique, or the prism coupling method should be used on single crystals that have been indexed, cut, and polished^10^. The PM wavelength range (and angles) are then determined experimentally using measured *n*(*λ*) dispersion relations fit to analytical Sellmeier expressions^11^. Owing to the nature of the optimization problem and optical dispersion, the refractive indices should be measured at a minimum of five different wavelengths. After obtaining the Sellmeier equations, numerical solutions to the appropriate PM-angle equations may be performed to find the optimal directions and PM wavelength ranges^12^.

The refractive indices can also be obtained from electronic structure calculations. Because the refractive index is attributable to a first-order perturbation of the ground state wave functions, high-precision calculations utilizing a complete basis set and high numerical tolerances are required. Careful convergence tests should also be performed, otherwise erroneous results may be obtained (Fig. 2). Limitations imposed by the computational method on the band-gap accuracy also extend to predictions on calculated birefringence values. Best practice is to report computationally obtained refractive indices at the level of theory consistent with the most accurate band gap. However, in cases where the experimental gap is known, ad hoc corrections to the gap could be made followed by self-consistent calculation of the refractive indices at the experimental gap. Finally, the nonlinear SHG material response should be determined from single-crystal measurements. Individual *d*_*ij*_ coefficients—SHG coefficients—may be measured using the Maker-Fringe technique^13^. For DUV applications, a *d*_*ij*_ > 0.39 pm/V at 1064 nm is required. Conversion efficiencies may also be reported provided authentic comparisons to known materials are performed.

The frequency-dependent and/or static *d*_*ij*_ values can also be calculated using perturbation theory, although in most cases it can be enough to report only the static response because most materials show negligible dispersion in the DUV. However, these optical responses are sensitive to the numerical approximations in the calculations. Important considerations are the number of empty conduction bands used in calculating the matrix elements for *d*_*ij*_ as variations up to 80% can occur if the number of empty states is insufficient^14^. The accuracy of the exchange-correlation functional in capturing the band gap and local chemical bonding environments should be assessed as the SHG response can vary on the order of 0.5 pm/V between local and nonlocal functionals. One should avoid choosing the functional based on which gives the highest value, but rather by understanding if the electronic and atomic structure descriptions of the material are well described.

It is best to provide as much experimental data about a new material as possible, as unsatisfactorily performed calculations can lead to erroneous conclusions about the capability of the DUV NLO material. If no experimental data are available for a predicted compound, the variations in the optical properties obtained from different levels of theory should be discussed to assess NLO performance.

### Additional considerations

A functional NLO crystal should be physically stable before, during and after operation, and their synthesis should not require any toxic or restricted-access reagents, for example. The mechanical, thermal, and environmental stability are also important factors to consider for material commercialization. The crystal should have a sufficiently high laser-damage threshold (LDT). For DUV NLO materials, a LDT > 5 GW/cm^2^ for a nanosecond pulse at 1064 nm is required.

## Outlook

We have summarized the minimum—but perhaps not all—of the important features to assess when determining whether a new DUV NLO material is technologically functional with a realistic chance of commercialization. The presented discovery and assessment workflow and feature checklist may be used to understand how to circumvent property dichotomies in optical materials; they may also be extended beyond the goal of realizing high-performing DUV NLO crystals to address challenges in photonics and light-based sciences across the electromagnetic spectrum.
